# Metal-Enhanced Fluorescence of Silver Island Associated with Silver Nanoparticle

**DOI:** 10.1186/s11671-016-1247-6

**Published:** 2016-01-16

**Authors:** Jiunn-Woei Liaw, Hsin-Yu Wu, Chu-Chuan Huang, Mao-Kuen Kuo

**Affiliations:** Department of Mechanical Engineering, Chang Gung University, 259 Wen-Hwa 1st Rd., Kwei-Shan, Taoyuan, 333 Taiwan; Center for Biomedical Engineering, Chang Gung University, 259 Wen-Hwa 1st Rd., Kwei-Shan, Taoyuan, 333 Taiwan; Medical Physics Research Center, Institute for Radiological Research, Chang Gung University and Chang Gung Memorial Hospital, Taoyuan, 333 Taiwan; Institute of Applied Mechanics, National Taiwan University, 1, Sec. 4, Roosevelt Rd., Taipei, 106 Taiwan

**Keywords:** Metal-enhanced fluorescence, Silver island, Silver nanoparticle, Surface plasmon resonance, Oblate spheroid, Excitation rate, Apparent quantum yield, Enhancement factor, MMP

## Abstract

The coupling plasmon of a hybrid nanostructure, silver island (SI) associated with silver nanoparticle (SNP), on metal-enhanced fluorescence (MEF) was studied theoretically. We used the multiple multipole method to analyze the plasmon-mediated enhancement factor on the fluorescence of a molecule immobilized on SNP and located in the gap zone between SI and SNP; herein, the SI was modeled as an oblate spheroid. Numerical results show that the enhancement factor of the hybrid nanostructure is higher than that of a SNP or a SI alone due to the coupled gap mode. This finding is in agreement with the previous experimental results. In addition, the plasmon band of the structure is broadband and tunable, which can be red-shifted and broadened by flattening or enlarging SI. Based on this property, the hybrid nanostructure can be tailored to obtain the optimal enhancement factor on a specific molecule according to its excitation spectrum. Moreover, we found that there is an induced optical force allowing SNP be attracted by SI. Consequently, the gap is reduced gradually to perform a stronger MEF effect.

## Background

In the past decade, using metallic nanostructures to perform the metal-enhanced fluorescence (MEF; or called surface-enhanced fluorescence) has attracted a lot of attentions [1–11]. Due to the localized surface plasmon resonance (SPR) of gold or silver nanostructures, the local electric field in their vicinity can be enhanced significantly to raise the excitation rate on a nearby molecule [11–17]. Additionally, the plasmon-mediated Forster resonance energy transfer (FRET) between the excited molecule and the nanostructure facilitates the emission of the fluorescence, so as to raise the quantum yield and reduce the lifetime of the fluorescence dramatically [6–8, 15–21]. In particular, for some molecular fluorescence with low quantum yield, the MEF becomes of importance [7]. The advantages of MEF include the increased detectability and photostability of fluorophores. Moreover, Purcell effect has elucidated that the environment can modify the spontaneous emission of emitters [22–24]. A variety of nanostructures have been proposed for the purpose such as silver nanoparticle (SNP), nanoshell, gold nanorod, silver nanotriangle, and silver island film (SIF) [25–40]. For example, SIF with discrete silver islands (SIs) on a substrate has been widely used for sensing single molecular fluorescence and protein in nano-biotechnology [2, 5, 8, 10]. Recently, a hybrid nanostructure using SIF associated with SNPs has been developed to enhance the fluorescence of molecules located within the gap zone between the SI and SNP [41]. The hybrid nanostructure of SNPs over SIF on a substrate forms a sandwiched layer where the detected molecule is in between the gap of SI and SNP. Within the nanogap, which is a hot spot, the MEF can be performed significantly. In addition, this new structure can also be applied to the surface-enhanced Raman scattering (SERS) [42–44].

In this paper, the MEF performance of the new hybrid nanostructure, a SI associated with a SNP, is studied and characterized theoretically [41]. In this hybrid nanostructure, SI associated with SNP forms a heterodimer with a nanogap [41, 45, 46]. We use the multiple multipole (MMP) method to analyze the excitation rate, apparent quantum yield, and enhance factor (EF) upon a molecule within the gap zone of the hybrid nanostructure [47–50]. Here, SI is simply modeled as an oblate spheroid. The MMP method has been used to study the MEF of a metallic dimer structure [23, 45, 46]. In this paper, we assume that the molecule is immobilized on the surface of SNP with a fixed distance for real applications. The effects of the gap between SI and SNP and the distance between molecule and SNP on the excitation rate are investigated.

## Methods

**Fig. 1:**
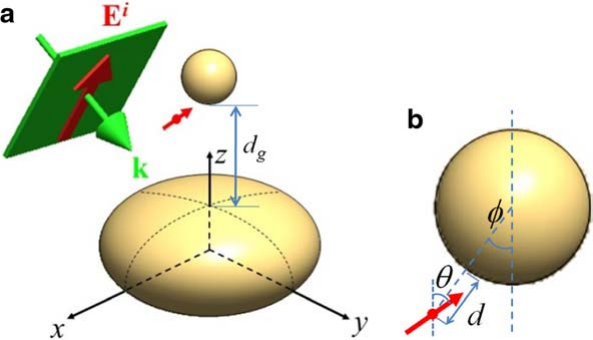
**a** Configuration of SI (an oblate spheroid), associated with SNP conjugated with a molecule on the surface, irradiated by an incident-polarized plane wave in water, where **k** and **E** ^*i*^ are on *yz* plane, the incident angle between **k** and −**e**_*z*_ is *α*, and the gap between SI and SNP is *d*_*g*_. **b** The enlarged drawing of SNP conjugated with a molecule, where the orientation angle of the dipole moment of molecule is *θ*, the distance between SNP and molecule is *d*, and the angle of molecular position *r.w.t.* the center of SNP is *ϕ*

The configuration of a SI with an oblate-spheroid shape of ${\left(\frac{x}{a}\right)}^2+{\left(\frac{y}{a}\right)}^2+{\left(\frac{z}{b}\right)}^2=1$ associated with a SNP above it is shown in Fig. 1a, where the distance of gap between SNP and SI is denoted by *d*_*g*_. The aspect ratio (AR) of SI is *a/b*; *a > b*. We assume that a molecule is immobilized on the surface of SNP with a distance *d*; the relative position *r.w.t.* SNP is shown in Fig. 1b. In the following, the MEF of the molecule located within the gap zone is particularly discussed. The wavenumber vector and the electric field of the incident *p*-polarized plane electromagnetic (EM) wave are denoted by **k** and **E**^*i*^, respectively; both vectors are assumed on the *yz* plane. The obliquely incident angle of the plane wave is denoted by *α*; the angle between **k** and **−e**_*z*_. In addition, the position vector of the molecule is represented by **x**_*d*_. The unit vector of the dipole moment **e**_*d*_ of the molecule (electric dipole) is also on the *yz* plane; **e**_*d*_ = (0, sin*θ*, cos*θ*), where *θ* is the angle between **e**_*d*_ and **e**_*z*_.

### Excitation Rate

For the excitation stage of the molecule, the hybrid nanostructure is irradiated by an incident plane EM wave. The excitation rate at an excitation wavelength *λ*_ex_, which is the normalized intensity for exciting the emitter, is defined as [51, 52]

1 $$ \Psi \left({\mathbf{e}}_d,{\mathbf{x}}_d;{\lambda}_{ex}\right)={\left|\mathbf{E}\left({\mathbf{x}}_d;{\lambda}_{ex}\right)\cdot {\mathbf{e}}_d\right|}^2/{\left|{\mathbf{E}}^i\right|}^2 $$

Here, **E** is the total electric field induced by the incident plane wave at the emitter’s location **x**_*d*_, and |**E**^*i*^| is the amplitude of the electric field of the incident plane wave. The total EM fields (**E**, **H**) in the exterior area are the linear sum of the incident and scattered fields: **E** = **E**^*i*^ + **E**^*s*^ and **H** = **H**^*i*^ + **H**^*s*^. According to the previous researches, the strongest electric fields always occur in the gap zone between metallic dimer [23, 45, 46]. Therefore, the hybrid nanostructure performs as a nanolens to focus an incident wave into the gap zone, which is a hotspot area [41]. The excitation rate at the position of the molecule (emitter) exhibits the amplification effect of the electric field caused by the hybrid nanostructure.

### Apparent Quantum Yield

Once the excited molecule is activated, it behaves as an oscillating electric dipole (an emitter) to radiate fluorescence at an emission wavelength *λ*_em_ in the subsequent emission stage. Since the radiation of the emitter is significantly affected by the hybrid nanostructure, the interaction of the dipole with the nearby SI and SNP is needed to be further studied. For this model, MMP method is also applied to analyze the near-field and far-field responses of the emission of the emitter. First, we need to calculate the dipole’s radiative power **P**_*r*_, the power emitted to the far field, and the nonradiative power **P**_*nr*_, the dissipating one inside the metallic hybrid nanostructure [23]. Both powers of the electric dipole with an orientation vector **e**_*d*_ at the position **x**_*d*_ in the presence of the hybrid nanostructure are expressed in terms of the Poynting vector ${\mathbf{E}}^d\times {\overline{\mathbf{H}}}^d$ in terms of the total EM fields (**E**^*d*^, **H**^*d*^) in the exterior area as,

2 $$ {\mathrm{P}}_r=\frac{1}{2}Re\left\{{\displaystyle \underset{S}{\int }{\mathbf{E}}^d\times {\overline{\mathbf{H}}}^d\cdot \mathbf{n}\kern0.1em ds}\right\} $$

3 $$ {\mathrm{P}}_{nr}=\frac{-1}{2}Re\left\{{\displaystyle \underset{S_c}{\int }{\mathbf{E}}^d\times {\overline{\mathbf{H}}}^d}\cdot \mathbf{n}\kern0.1em ds\right\} $$

where *S* is an any simply closed surface enclosing the dipole and the hybrid nanostructure and *S*_*c*_ is the total surface of SI and SNP [23, 51, 52]. Here, *Re* denotes the real part, and the over bar is the complex conjugate. In the following, these two powers will be normalized by the radiative power of a free electric dipole. We assume the surrounding medium is lossless material, e.g., water, and the molecule is an ideal emitter. In terms of the two powers, the apparent quantum yield *η* of the system is defined by

4 $$ \eta \left({\mathbf{e}}_d,{\mathbf{x}}_d;{\lambda}_{em}\right)=\frac{{\mathrm{P}}_r}{{\mathrm{P}}_r+{\mathrm{P}}_{nr}}. $$

The apparent quantum yield represents the efficiency of the emission of an ideal electric dipole affected by a nearby hybrid nanostructure; 0 ≤ *η* ≤ 1 [23].

### Enhancement Factor

Furthermore, the EF is defined as a multiple of the excitation rate and the apparent quantum yield, Ψ(**e**_*d*_, **x**_*d*_; *λ*_*ex*_) ⋅ *η*(**e**_*d*_, **x**_*d*_; *λ*_*em*_), where *λ*_*ex*_ and *λ*_*em*_ are the excitation and emission wavelengths, respectively [23]. Here, EF is a function of **e**_*d*_ and **x**_*d*_, as well as *λ*_*ex*_ and *λ*_*em*_; *λ*_*ex*_ ≤ *λ*_*em*_. In addition, EF depends on the configuration of the plasmonic hybrid nanostructure and the incident angle of the plane wave, as shown in Fig. 1.

### Optical Force

When the hybrid nanostructure is irradiated by the incident plane wave, optical forces exerted upon SNP and SI are also induced. Since the SI is assumed to be fixed on a substrate, we only consider the optical force on SNP in the following. The optical force **F** in terms of Maxwell stress tensor **T** can be expressed by a surface integral,

5 $$ \mathbf{F}={\displaystyle \underset{S_{NP}}{\int}\mathbf{T}\cdot \mathbf{n}ds} $$

where *S*_*NP*_ is the surface of SNP. The Maxwell stress tensor in terms of the total EM field is expressed as [53, 54]

6 $$ \mathbf{T}=\frac{1}{2}Re\left\{\varepsilon \mathbf{E}\overline{\mathbf{E}}+\mu \mathbf{H}\overline{\mathbf{H}}-\frac{1}{2}\left(\varepsilon \mathbf{E}\cdot \overline{\mathbf{E}}+\mu \mathbf{H}\cdot \overline{\mathbf{H}}\right)\;\mathbf{I}\right\} $$

which is the average value of a period**.**

## Results and discussion

**Fig. 2:**
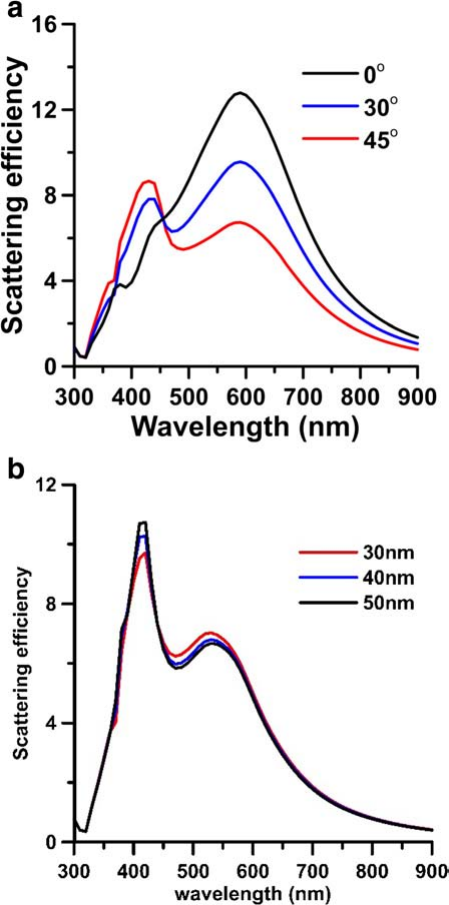
Scattering efficiencies of a hybrid nanostructure consisting of **a** a SI of (70, 70, 35) nm associated with a SNP of *r* = 30 nm, with a gap of 30 nm irradiated by plane waves with different incident angles (*α =* 0°, 30°, and 45°), and **b** a SI of (60, 60, 35) nm associated with a SNP of *r* = 30 nm with different gap *d*_*g*_ (30 nm, 40 nm or 50 nm) irradiated by 45°-incident plane wave

A typical SI of (*a*, *a*, *b*) = (70, 70, 35) nm with an AR of *a/b* = 2 is used for study. The surrounding medium is water, and the frequent-dependent permittivity of silver in Ref. [55] is used for analysis. Figure 2a shows the scattering efficiencies of a hybrid nanostructure, a SI of (70, 70, 35) nm associated with a SNP of 30 nm, with a gap of 30 nm irradiated by plane waves with different incident angles (*α =* 0°, 30° and 45°) [56]. From these spectra, the plasmon band of this hybrid nanostructure is broad, from 400 to 650 nm, where the plasmon peak is at 593 nm. The results demonstrate that the longitudinal plasmon band of SI (60, 60, 35) nm is also wide and the plasmon-peak wavelength is 562 nm, which is shorter than that of SI (70, 70 35) nm. This property illustrate that the plasmon band of the structure is tunable by tailoring the AR of SI. In Fig. 2a, the first mode at 420 nm is corresponding to the plasmon mode of SNP as well as the transverse plasmon mode of SI and the second one at 593 nm the longitudinal plasmon mode of SI; the transverse mode and longitudinal mode are the results of the collective motion of free electrons oscillating along the short and long axes, respectively. When the incident angle is 0° (the normal incidence case), the electric field of the incident light is parallel to the long axis (*y* axis), so the longitudinal plasmon mode of SI is easily induced. Hence, as the incident angle increases, the contribution of this longitudinal mode will be reduced, and that of transverse mode will be increased. In addition, the scattering efficiencies of another hybrid nanostructure with a small SI of (60, 60, 35) nm are shown in Fig. 2b for different gaps (30, 40, 50) nm; AR = 1.71. Figure 2b shows that these two modes are at 420 and 540 nm, almost the same for different gaps (30, 40, 50) nm. This is because the coupling modes of the hybrid nanostructure do not clearly appear until that the gap is smaller than 10 nm.

**Fig. 3:**
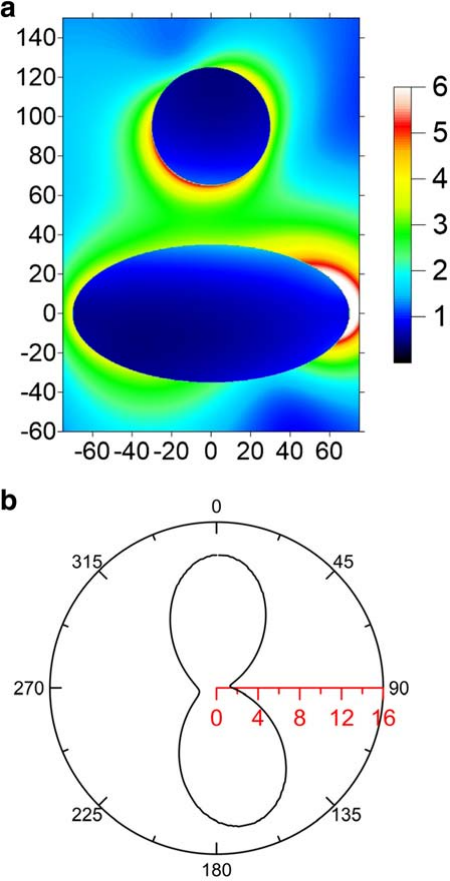
**a** Near field |E| and **b** far-field scattering pattern in *yz* plane for SI of (70, 70, 35) nm coupled with SNP of *r* = 30 nm irradiated by a 45°-incident plane wave of 593 nm in water, where *d*_*g*_ = 30 nm

**Fig. 4:**
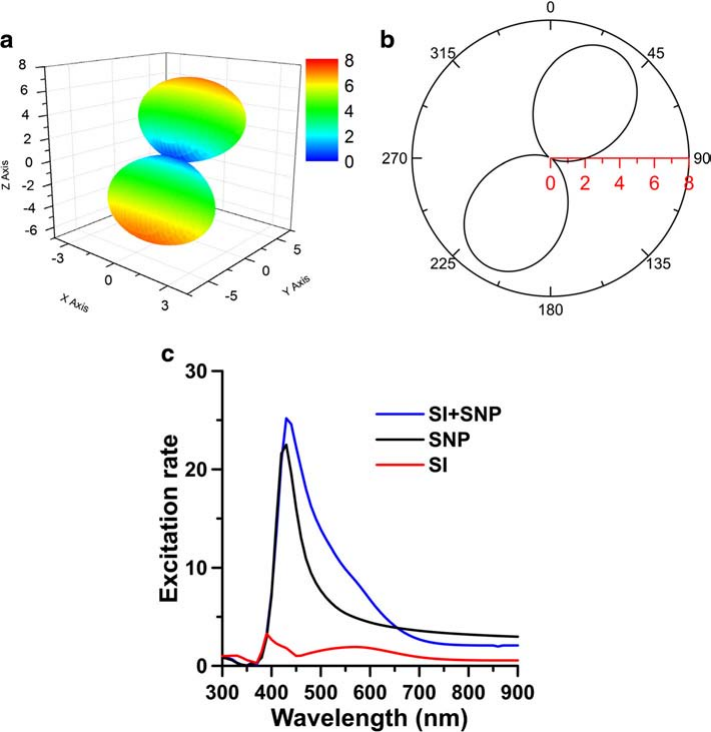
**a** 3D spherical plot of excitation rate versus the orientation angle *θ* of electric dipole affected by SI of (70, 70, 35) nm associated with SNP of *r* = 30 nm. **b** The *yz-*plane cross section of **a**, where *d*_*g*_ = 30 nm, *d* = 15 nm, *λ*_*ex*_ = 593 nm, and *α* = 45°. **c** Excitation rates of hybrid nanostructure (SI + SNP), SI or SNP alone versus excitation wavelength *λ*_*ex*_, where *θ* = 31°

**Fig. 5:**
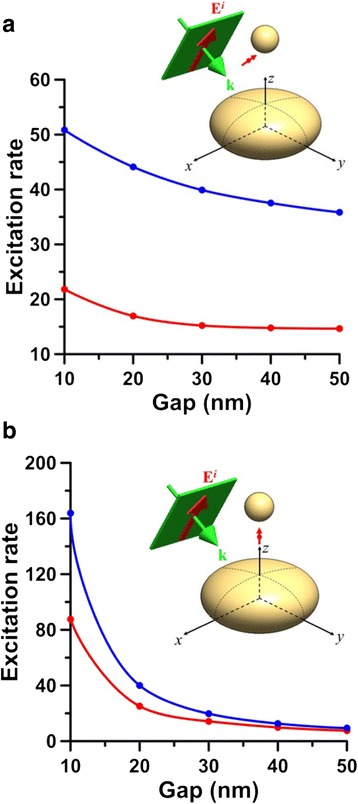
Excitation rates versus gap *d*_*g*_ induced by 45°-incident plane wave of 593 nm (*red*) or 488 nm (*blue*) upon differently located molecules with **a** *ϕ* = 45° and *θ* = 31° and **b** *ϕ* = 0° and *θ* = 0°. SI of (70, 70, 35) nm, SNP: *r* = 30 nm, and *d* = 5 nm

The *yz*-plane cross section of the near-field |E| distribution and the far-field scattering pattern $Re\left({\mathbf{E}}^s\times {\overline{\mathbf{H}}}^s\cdot {\mathbf{e}}_R\right){R}^2$ as *R* → ∞ of the hybrid nanostructure consisting SI of (70, 70, 35) nm and SNP of *r* = 30 nm are shown in Fig. 3, where the incident angle is 45° and the excitation wavelength is 593 nm. Here, *R* is the distance of the observing point from the origin of the coordinates. The scattering pattern demonstrates that the directionality of the scattered Poynting vector (energy flux) at far field is along the *z* axis. This is because that the plasmon oscillation along the long axis (*y* axis) of SI dominates the light scattering, no matter of the incident angle. Therefore, the far-field scattering cross section looks like an 8-shape with a major energy flux along the *z* axis. Furthermore, we assume the molecular position is at *ϕ* = 45° with a distance *d* = 15 nm from SNP, as shown in Fig. 1b. For this case, the 3D spherical plot of the excitation rate Ψ(**e**_*d*_, **x**_*d*_; *λ*_*ex*_) versus the orientation angle *θ* of the electric dipole is shown in Fig. 4a, where *λ*_*ex*_ = 593 nm and *α* = 45°. The *yz*-plane cross section of Fig. 4a is shown in Fig. 4b; the maximum excitation rate occurs at *θ* = 31°. Subsequently, the excitation rate of the hybrid nanostructure versus the excitation wavelength is shown in Fig. 4c, where *α* = 45°, *d*_*g*_ = 30 nm, *d* = 15 nm, and *θ* = 31°. The excitation rates of SI or SNP alone are also plotted in Fig. 4c. Comparing these curves, we can find that the excitation rate of the hybrid nanostructure is larger than that of SI or SNP alone. Additionally, the excitation-rate spectra of the hybrid nanostructure are broadband, compared to those of SI and SNP alone. The maximum excitation rate is 25 at 440 nm, and the excitation rate is still as high as 7.24 at 593 nm. We can increase the excitation rate by reducing the gap. Figure 5a shows that the excitation rate versus the gap *d*_*g*_ at *λ*_*ex*_ = 488 or 593 nm, where *d* = 5 nm, *ϕ* = 45°, and *θ* = 31°. The results indicate that the excitation rate of 488 nm is larger than that of 593 nm and can be increased as the gap is reduced. However, if the distance between molecule and nanostructure is too small, the apparent quantum yield could be reduced dramatically to cause the quenching of fluorescence [48, 49]. Moreover, if the molecule is located at the center line of the hybrid nanostructure (i.e., *ϕ* = 0°), the excitation rate can be raised more, as shown in Fig. 5b, where *d* = 5 nm and *θ* = 0°. This illustrates that the excitation rate is also sensitive to the molecular location in the gap zone.

**Fig. 6:**
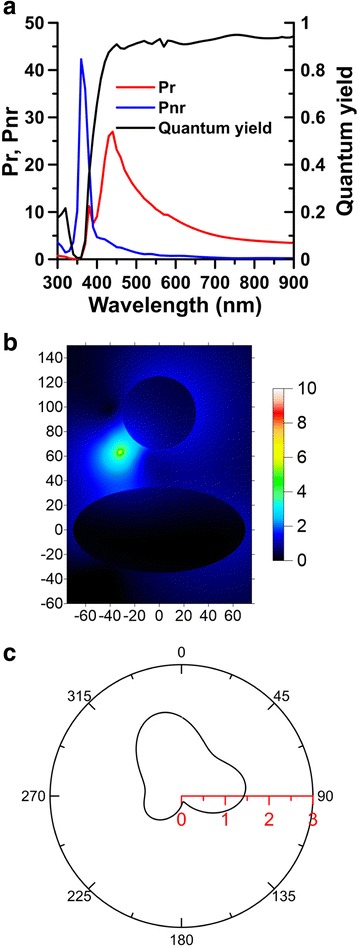
**a** Radiative power, nonradiative power, and apparent quantum yield versus emission wavelength. **b** Near-field electric field distribution. **c** Far-field radiation pattern in *yz* plane of an electric dipole with *θ* = 31° at *λ*_*em*_ = 618 nm affected by a SI of (70, 70, 35) nm associated with SNP of *r* = 30 nm, where *d*_*g*_ = 30 nm, *ϕ* = 45°, and *d* = 15 nm

Figure 6a shows the normalized radiative and nonradiative powers, and the apparent quantum yields of an emitter with *θ* = 31° affected by a hybrid nanostructure, SI of (70, 70, 35) nm associated with SNP of *r* = 30 nm, where *d*_*g*_ = 30 nm, *ϕ* = 45°, and *d* = 15 nm. These results indicate that the plasmon-mediated FRET can enhance the radiative and nonradiative powers both within a broadband range of 400 to 700 nm. This implies that the lifetime of fluorescence in this range can be reduced dramatically. Moreover, the apparent quantum yield indicates that the hybrid nanostructure is like a low-pass filter with a cutoff wavelength of 400 nm for the emission of a vicinal electric dipole. When the emission wavelength is longer than 400 nm, the apparent quantum yield is about 0.9. In contrast, as the emission wavelength is shorter than 400 nm, the emission is severely suppressed. The near-field mapping of |E| of an electric dipole at *λ*_*em*_ = 618 nm is shown in Fig. 6b. The far-field radiation pattern in *yz* plane of an electric dipole in the presence of the hybrid nanostructure is plotted in Fig. 6c; $Re\left({\mathbf{E}}^d\times {\overline{\mathbf{H}}}^d\cdot {\mathbf{e}}_R\right){R}^2$ as *R* → ∞ at *λ*_*em*_ = 618 nm. The far-field radiative pattern demonstrates that the emission on the backside of SI is suppressed, and the main lobe of the radiation pattern is in the opposite direction of the incident plane wave, as shown in Fig. 6c. This important directionality of the emission is attributed to the nanoantenna effect of the hybrid nanostructure.

**Figure 7:**
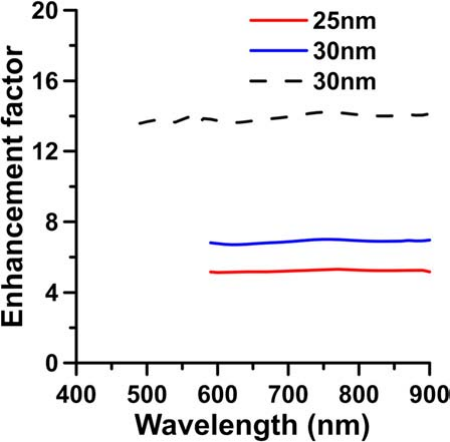
EF versus emission wavelength for a SI of (70, 70, 35) nm associated with SNP of *r* = 25 nm or 30 nm on an emitter with *θ* = 31° and a distance *d =* 15 nm from SNP, where *ϕ* = 45°, *α* = 45°, *d*_g_ = 30 nm, and *λ*_*ex*_ = 488 nm (*dash line*) or 593 nm (*solid lines*)

In order to assess the overall MEF effect of the hybrid nanostructure on the spontaneous emission of a single emitter, the EF, Ψ(**e**_*d*_, **x**_*d*_; *λ*_*ex*_) ⋅ *η*(**e**_*d*_, **x**_*d*_; *λ*_*em*_), is calculated. Figure 7 shows the EF for a SI of (70, 70, 35) nm associated with SNP of *r* = 25 or 30 nm on an emitter with an orientation of *θ* = 31° and a distance *d =* 15 nm from SNP versus the emission wavelength *λ*_*em*_, where *ϕ* = 45°, *d*_g_ = 30 nm, *α* = 45°, and *λ*_*ex*_ = 593 nm (solid lines) or 488 nm (dash line). Due to the Stokes shift of fluorescence, *λ*_*em*_ is longer than *λ*_*ex*_. The EF of the hybrid nanostructure is 6.74 for the fluorescence of a specific molecule (e.g., Texas Red) excited at 593 nm and emitting at 618 nm for SNP of *r* = 30 nm, and EF is 14 for another molecule (e.g., fluorescein isothiocyanate (FITC)) excited at 488 nm and emitting at 520 nm. For a smaller SNP of 25 nm, the EF of the hybrid nanostructure is 5.2 at *λ*_*ex*_ = 593 nm and *λ*_*em*_ = 618 nm. The previous experimental results have shown that EF for a molecule can be as high as 60-fold at *λ*_*ex*_ = 635 nm and *λ*_*em*_ = 670 nm in air, where the distance *d* between molecule and SNP is 5 nm and the gap *d*_*g*_ between SNP and SI is 10 nm [41]. According to our study, we can raise the excitation rate to increase the EF by reducing *d* and *d*_*g*_. For example, if the gap *d*_*g*_ is reduced to 10 nm and *d* = 5 nm, the EF can be raised to 69 for *λ*_*ex*_ = 593 nm and *λ*_*em*_ = 618 nm and to 128 for *λ*_*ex*_ = 488 nm and *λ*_*em*_ = 520 nm according to the excitation rate shown in Fig. 5b. Here, we assume the apparent quantum yield for emission is 0.8.

**Fig. 8:**
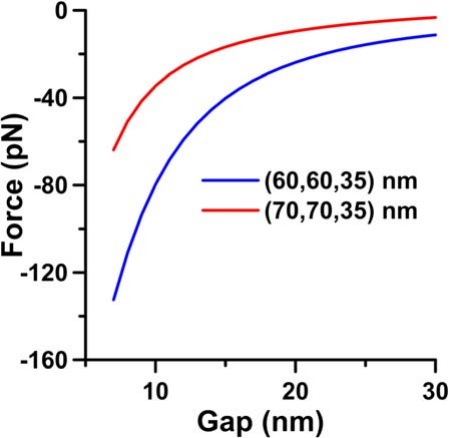
The *z-*component of optical force driving SNP of *r* = 30 nm toward SI of (70, 70, 35) nm or (60, 60, 35) nm versus gap *d*_*g*_ induced by a 45°-incident plane wave of 593 nm

Furthermore, we studied the attraction effect of optical force induced by the irradiance of the incident plane wave. Figure 8 shows the *z-*component of optical forces exerted on SNP of *r* = 30 nm versus gap *d*_*g*_ induced by a 45°-incident plane wave of 593 nm, where the size of SI is (70, 70, 35) nm or (60, 60, 35) nm. These curves in Fig. 8 indicate that the performance of the optical force is attraction; the optical force drives SNP toward SI. In addition, the smaller the gap the larger the attractive force is. Here, the fluence of the light is assumed 25 MW/cm^2^. Actually, the amplitude of the optical force is linearly proportional to the fluence of the incident plane wave. Our finding demonstrates that the induced optical force could gradually make the gap between SNP and SI smaller. As a result, the EF of the hybrid nanostructure will increase as the irradiance time and fluence of laser beam increase. Of course, the optical force needs to be large enough to overcome the Brownian motion; otherwise, the attraction phenomenon cannot be observed. Our finding is in agreement with the previous report that SERS signal was increased dramatically by using optical tweezers to aggregate SNPs [57].

## Conclusions

The wavelength-dependent MEF of a hybrid nanostructure, a SI associated with a SNP, upon a molecule located in the gap zone was studied theoretically by MMP method, where the SI was modeled as an equivalent oblate spheroid. The excitation rate and the apparent quantum yield of the molecule affected by the hybrid nanostructure were analyzed quantitatively. In terms of the two factors, the EF of the hybrid nanostructure, depending on the excitation and emission wavelengths, was evaluated quantitatively. Numerical results illustrate that the excitation rate for a molecule, immobilized on SNP and located in the gap zone between SI and SNP, at the excitation stage very depends on the gap size and the distance from SNP; the smaller the gap and distance, the larger the excitation rate. In the excitation stage, the hybrid nanostructure performs as a nanolens to focus the incident plane wave into the gap zone to induce a hot spot. In addition, the excitation-rate spectrum is a broadband one, because the hybrid nanostructure’s plasmon band is wide; the plasmon band of the structure can be red-shifted and broadened by increasing the AR of SI. On the other hand, the hybrid nanostructure performs as a low-pass filter for the emission of an emitter (an excited molecule), with a cutoff wavelength of 400 nm. As a result, the EF of the hybrid nanostructure on a fluorescent molecule is also broadband. If the excitation spectrum of a specific molecule is within the range of 400 to 650 nm, this hybrid nanostructure can perform a remarkable MEF. Based on these properties, this hybrid nanostructure can be tailored to obtain the optimal enhancement factor on a specific molecule according to its excitation spectrum. For example, when the gap *d*_*g*_ between SI and SNP is 30 nm and the distance *d* between SNP and molecule is 15 nm, the EF is 6.74 for the fluorescence of a specific molecule (e.g., Texas Red) excited at 593 nm and emitting at 618 nm. For another molecule (e.g., FITC), the EF is 14 as it is excited at 488 nm and emits at 520 nm. Our study also indicates that to raise the EF, we need to decrease the distance between molecule and SNP as well as to reduce the gap between SNP and SI. For example, if the gap *d*_*g*_ is reduced to 10 nm and *d* = 5 nm, the EF can be raised to 69 for *λ*_***ex***_ **=** 593 nm and *λ*_***em***_ **=** 618 nm and to 128 for *λ*_***ex***_ **=** 488 nm and *λ*_***em***_ **=** 520 nm. The EF of the hybrid nanostructure is also dependent on the location of the molecule. In general, as the molecule is close to the center line of SNP and SI as well as close to the surface of SNP, the EF is increased. In addition, the hybrid nanostructure plays another important role of a nanoantenna to guide the directionality of the molecular emission. Moreover, we found that the optical force induced by the incident light can drive the SNP approach SI to reduce the gap for obtaining a stronger MEF effect. Our finding might pay a way to the applications of using the sandwiched hybrid nanostructure for MEF on single molecular fluorescence. Recently, using thermal annealing to transform AgOx thin film into a SIF was proposed, where two adjacent SIs form a dimer with a small gap to provide the hotspot for the SERS of R6G and MEF of different dyes due to the broadband performance [58]. Hence, it is prospective to combine of this SIF with additive SNPs for the applications in MEF and SERS.

## Abbreviations

AR: aspect ratio
EF: enhancement factor
EM: electromagnetic
FRET: Forster resonance energy transfer
MEF: metal-enhanced fluorescence
MMP: multiple multipole
SERS: surface-enhanced Raman scattering
SI: silver island
SIF: silver island film
SNP: silver nanoparticle
SPR: surface plasmon resonance
